# The association between pre‐diagnostic levels of psychological distress and adverse effects after radical prostatectomy

**DOI:** 10.1002/bco2.334

**Published:** 2024-02-24

**Authors:** Rasmus Nilsson, Thomas Næss‐Andresen, Tor Åge Myklebust, Tomm Bernklev, Hege Kersten, Erik Skaaheim Haug

**Affiliations:** ^1^ Department of Urology Telemark Hospital Trust Skien Norway; ^2^ Institute of Clinical Medicine, Faculty of Medicine University of Oslo Oslo Norway; ^3^ Department of Surgery, Division of Urology Vestre Viken Hospital Trust Drammen Norway; ^4^ Department of Registration Cancer Registry Norway Oslo Norway; ^5^ Department of Research and Innovation Møre and Romsdal Hospital Trust Ålesund Norway; ^6^ Department of Research and Innovation Vestfold Hospital Trust Tønsberg Norway; ^7^ Department of Research Telemark Hospital Trust Skien Norway; ^8^ Department of Urology Vestfold Hospital Trust Tønsberg Norway; ^9^ Institute for Cancer Genomics and Informatics Oslo University Hospital Oslo Norway

**Keywords:** anxiety, depression, erectile dysfunction, prostate cancer, radical prostatectomy, urinary incontinence

## Abstract

**Objectives:**

To prospectively analyse the associations between pre‐diagnostic levels of anxiety and depression and patient‐reported urinary and sexual adverse effects after radical prostatectomy in a population‐based setting.

**Patients and Methods:**

In three Norwegian county hospitals, men referred with a suspicion of prostate cancer were asked to fill out a patient‐reported outcome measurement (PROM) questionnaire prior to prostate biopsy. Those who later underwent radical prostatectomy were stratified into three distress groups according to their Hopkins Symptom Checklist 5‐score. Additional PROM questionnaires, including the EPIC‐26 to measure adverse effects, were collected at 6 and 12 months postoperatively. Multivariable mixed models were estimated and post hoc pairwise comparisons performed to explore differences in adverse effects between distress groups.

**Results:**

A total of 416 men were included at baseline and of those, 365 (88%) returned questionnaires at 6 months and 360 (87%) at 12 months. After adjusting for confounders, men with high distress at baseline had worse urinary incontinence domain score (58.9 vs. 66.8, *p* = 0.028), more urinary bother (64.7 vs. 73.6, *p* = 0.04) and a higher risk of using incontinence pads (70.6% vs. 54.2%, *p* = 0.034) at 6 months than those with low distress. There was no difference in the sexual domain scores between distress groups postoperatively, but the high‐distress group expressed more sexual bother (24.9 vs. 37.5, *p* = 0.015) and the intermediate‐distress group had a greater probability of using sexual medications or devices (63.8% vs. 50.0%, *p* = 0.015) than the low‐distress group at 6 months. At 12 months scores generally improved slightly and differences between distress groups were less evident.

**Conclusion:**

Men with higher levels of anxiety and depression before prostate biopsy report more urinary and sexual adverse effects after radical prostatectomy. This should be considered both in treatment decision‐making and during follow‐up after radical prostatectomy.

## 1 INTRODUCTION

The prevalence of urinary and sexual adverse effects (AEs) after radical prostatectomy (RP) varies between studies and depends on patient selection and on how AEs are defined.^1^, ^2^, ^3^, ^4^, ^5^ Known associated factors include age, preoperative function, body mass index (BMI), comorbidity, cancer stage, and surgical technique.^1^, ^4^, ^5^, ^6^, ^7^, ^8^ AEs can have a great impact on patients' health‐related quality of life and outcome satisfaction after RP.^9^, ^10^ Lately, there has been an increased interest in possible associations between AEs and psychological factors.

An American longitudinal study showed that the presence of depressive symptoms before radical treatment for prostate cancer (PCa) was a predictor for more urinary and sexual dysfunction 6 months after treatment.^11^ Another study reported that increased anxiety and depression scores before RP were associated with worse urinary and sexual function at follow‐up.^12^ An Italian study found that RP patients with baseline depression less often had recovery of their erectile function at follow‐up, and recently, in a large German study, it was found that RP patients with high preoperative levels of anxiety and depression more often used incontinence pads and pro‐erectile drugs at follow‐up.^13^, ^14^

The above studies have some methodological weaknesses, that is, retrospective designs, high dropout‐rates, unmeasured confounders and a baseline measure of psychological distress just before surgery, which may not reflect the patient's general level of distress.^12^, ^15^ Furthermore, they were conducted at referral tertiary centres and may not be generalizable to a broader population.

Regardless of treatment, men with localized PCa have a long life expectancy and AEs may persist and even increase after many years.^16^, ^17^, ^18^ It is therefore important to identify risk factors for developing AEs in order to aid patient counselling and treatment decision‐making. The aim of this study was to investigate the association between pre‐diagnostic levels of anxiety and depression and urinary and sexual AEs after RP in a population‐based setting.

## 2 PATIENTS AND METHODS

### 2.1 Design and participants

This prospective observational study was conducted at the three county hospitals of Telemark, Vestfold and Vestre Viken in Norway. From December 2016 to June 2022, men referred with a suspicion of PCa were asked to participate in a broader study investigating the impact of psychological factors on outcomes in PCa treatment. Those who accepted completed a patient‐reported outcome measurement (PROM) questionnaire prior to prostate biopsy. Participants who after diagnosis underwent immediate RP were followed up with PROM questionnaires at 6 and 12 months. A sub‐analysis on the use of medications was performed on all participants from Telemark (*n* = 203).

### 2.2 Primary outcome variable

AEs were measured with the Expanded Prostate Cancer Index Composite (EPIC)‐26, where urinary, bowel, sexual and hormonal domain scores were calculated in accordance with Szymanski's original instructions.^19^ The range is 0–100, lower scores means more AEs. Minimal important difference (MID) ranges for the urinary incontinence (6–9 points), irritation/obstruction (5–7 points) and sexual (10–12 points) domains have previously been defined by Skolarus et al.^20^

In addition to domain scores, individual items of the EPIC‐26 describe pad use (Item 3), quality of erections (Item 9) and how bothersome the urinary (Item 5) and sexual (Item 12) function/lack of function are experienced (urinary and sexual bother). The Likert scales of these items were in accordance with the scoring instructions, converted to continuous scales (0–100) where lower scores mean more bother, more pad use and worse quality of erections. Furthermore, Item 3 was dichotomized into any use of pads (yes/no) and Item 9 into erection firm enough for intercourse (yes/no). Information on use of sexual aids was obtained with the question ‘Have you used any medications or devices to aid or improve erections? (yes/no)’.

### 2.3 Other scales and variables

The Hopkins Symptom Checklist 5 (HSCL‐5) was used to measure levels of anxiety and depression. The HSCL‐5 mean score (range 1–4) was in the present study referred to as psychological distress, which is in accordance with previous publications.^21^, ^22^ A cut‐off at HSCL = 2 has been proposed to identify persons with possible mental disorders.^23^ In our study, patients were divided into three groups according to their HSCL‐5‐score: low (HSCL‐5 = 1), intermediate (HSCL‐5 = 1.2–1.8) and high (HSCL‐5 ≥ 2.0) distress. Information on physical activity and comorbidity were collected with additional questions used in previous studies and relevant clinical data from medical records.^24^, ^25^

### 2.4 Statistical analyses

Descriptive statistics are presented as the frequency and percentage for categorical variables and as the mean and standard deviation (SD) for continuous variables. Univariate (unadjusted) comparisons were made using chi‐square tests for categorical variables and one‐way analysis of variance (ANOVA) for continuous variables. To model the longitudinal structure of the data, and to adjust for potential confounding, linear mixed models were estimated. The models included psychological distress (categorized), follow‐up time and the following a priori selected potential confounders: age, educational level, BMI, level of physical activity, comorbidity and PSA at biopsy (iPSA). The models also included interaction effects between distress and follow‐up time. Predicted outcomes were calculated from the estimated models, for each combination of distress and follow‐up time, fixing the other covariates to their respective means in the total sample. Results were plotted with corresponding 95% confidence intervals (CIs), and *p* values from pairwise comparisons were provided in a separate table using Wald tests.

### 2.5 Ethics

The Regional Committee for Medical and Health Science Research of South‐East Norway approved the study (#2016/925). Participants were included after giving written informed consent.

## 3 RESULTS

### 3.1 Univariate analyses

In the study period, 2562 men were invited to participate and 2162 (84%) accepted the invitation. Out of these, 436 underwent an immediate RP, whereof 416 had completed HSCL‐5 correctly at baseline and could be included in the analysis. At follow‐up, 365 (88%) returned the questionnaire at 6 months and 360 (87%) at 12 months. Attrition analysis showed that the 19 (5%) men who refrained from returning any postoperative questionnaires had a higher iPSA compared with the responders (15.5 vs. 10.0 μg/L, *p* = 0.001). Otherwise, there were no statistically significant differences regarding HSCL‐5‐score, age, level of education, physical activity, adjuvant treatment or EPIC domain scores (Table S1). At 12 months, the dropout rate was higher in the high‐distress group (27% vs. 12% and 11%, *p* = 0.004, Table 2). Table 1 displays the demographic and clinical variables at baseline, stratified according to distress group. The high‐distress group had significantly lower educational level than the low‐ and intermediate‐distress groups. No other statistically significant differences were found between groups. Table 2 shows the unadjusted findings from the urinary and sexual domains from the EPIC‐26. Compared with the low‐distress group, the intermediate‐ and high‐distress groups had inferior incontinence and irritation/obstruction scores and more urinary and sexual bother before diagnosis and the differences persisted at follow‐up, although not statistically significant at all time points. Fifty‐nine per cent of the sample had erection firm enough for intercourse at baseline and few kept their erectile function postoperatively, with no differences between the distress groups. Sixteen per cent had been using sexual medication or devises at baseline with no statistically significant differences between distress groups while at follow‐up, the intermediate group had significantly higher use.

**TABLE 1:** Unadjusted comparison of demographic and clinical variables between different distress groups of men undergoing radical prostatectomy (chi‐square tests for categorical variables and one‐way analysis of variance (ANOVA) for continuous variables).

Variables	Low distress (HSCL‐5 = 1) *N* = 196	Intermediate distress (HSCL‐5 = 1.2–1.8) *N* = 160	High distress (HSCL‐5 ≥ 2) *N* = 60	*p* value
Age (years) at diagnosis, mean (SD)	67.5 (6.4)	66.2 (6.3)	65.7 (5.8)	0.051
Level of education, *N* (%)				**0.024**
≤12 years	115 (59)	86 (54)	44 (75)	
>12 years	79 (41)	73 (46)	15 (25)	
BMI (kg/m^2^), mean (SD)	27	27	27.1	0.98
Physical activity, *N* (%)				0.63
Inactive	20 (10)	17 (11)	8 (13)	
Minimally active	87 (45)	61 (38)	21 (35)	
Highly active	88 (45)	81 (51)	31 (52)	
Comorbidities, *N* (%)				0.22
0–1	147 (75)	122 (76)	39 (65)	
2 or more	49 (25)	38 (24)	21 (35)	
PSA at biopsy (μg/L), mean (SD)	9.8 (5.6)	10.6 (9.8)	10.8 (7.8)	0.5
ISUP on prostate biopsy, *N* (%)				0.41
1	12 (6)	9 (6)	2 (3)	
2	58 (30)	37 (23)	12 (20)	
3	66 (34)	64 (40)	22 (37)	
4	42 (21)	30 (19)	13 (22)	
5	17 (9)	20 (12)	11 (18)	
Unilateral or bilateral nerve‐sparing surgery, *N* (%)	116 (59)	91 (57)	31 (57)	0.89
Pathological stage, *N* (%)				0.42
pT2	97 (49)	88 (43)	28 (47)	
pT3	99 (51)	92 (57)	32 (53)	
Postoperative radiotherapy, *N* (%)	18 (9)	14 (9)	7 (12)	0.78
Postoperative hormonal treatment, *N* (%)	14 (7)	13 (8)	5 (8)	0.92

*Note*: Statistically significant differences (*p* < 0.05) are highlighted with bold typeface.

**TABLE 2:** Unadjusted comparisons of EPIC variables between distress groups at different time points before and after radical prostatectomy (chi‐square tests for categorical variables and one‐way analysis of variance [ANOVA] for continuous variables).

Variables	Low distress (HSCL‐5 = 1)	Intermediate distress (HSCL‐5 = 1.2–1.8)	High distress (HSCL‐5 ≥ 2)	*p* value
Number of respondents, *N* (%)				
Before biopsy	196 (100)	160 (100)	60 (100)	
6 months postoperatively	170 (87)	143 (89)	52 (87)	0.72
12 months postoperatively	176 (89)	140 (88)	44 (73)	**0.004**
Urinary incontinence score, mean (SD)				
Before biopsy	94.2 (11.2)	89.8 (15.2)	89.6 (15.8)	**0.004**
6 months postoperatively	67.0 (27.8)	65.2 (27.4)	58.0 (26.1)	0.12
12 months postoperatively	70.9 (26.6)	69.7 (26.3)	63.1 (26.1)	0.22
Urinary irritation/obstruction score, mean (SD)				
Before biopsy	86.1 (13.1)	79.7 (16.9)	78.2 (19.0)	**<0.001**
6 months postoperatively	90.2 (12.3)	86.6 (12.8)	85.0 (12.6)	**0.008**
12 months postoperatively	90.1 (12.7)	87.4 (11.3)	87.1 (13.9)	0.11
Urinary bother score, mean (SD)				
Before biopsy	82.1 (23.8)	69.8 (30.2)	71.6 (30.9)	**<0.001**
6 months postoperatively	75.2 (28.1)	68.6 (29.6)	65.2 (29.6)	**0.042**
12 months postoperatively	75.1 (27.6)	70.5 (27.0)	67.0 (29.4)	0.14
Use of one or more pads per day, *N* (%)				
Before biopsy	4 (2)	5 (3)	2 (3)	0.74
6 months postoperatively	95 (56)	82 (58)	38 (73)	0.09
12 months postoperatively	74 (43)	65 (46)	24 (55)	0.35
Sexual domain score, mean (SD)				
Before biopsy	67.1 (27.7)	60.9 (27.5)	60.2 (27.3)	0.07
6 months postoperatively	17.9 (18.7)	17.7 (17.3)	17.0 (19.2)	0.95
12 months postoperatively	21.7 (20.6)	20.9 (19.3)	19.5 (20.9)	0.8
Sexual bother score, mean (SD)				
Before biopsy	73.2 (30.6)	63.9 (32.3)	59.3 (31.1)	**0.002**
6 months postoperatively	37.9 (34.7)	31.9 (31.8)	24.5 (31.1)	**0.028**
12 months postoperatively	41.2 (37.0)	35.9 (30.6)	27.8 (32.4)	0.053
Erection firm enough for intercourse, *N* (%)				
Before biopsy	118 (62)	87 (55)	37 (63)	0.44
6 months postoperatively	8 (5)	4 (3)	3 (6)	0.58
12 months postoperatively	11 (6)	7 (5)	2 (5)	0.84
Have been using sexual meds or aids, *N* (%)				
Before biopsy	27 (14)	30 (19)	9 (16)	0.44
6 months postoperatively	80 (49)	90 (64)	29 (56)	**0.029**
12 months postoperatively	88 (52)	84 (61)	18 (43)	0.07

*Note*: Statistically significant differences (*p* < 0.05) are highlighted with bold typeface.

### 3.2 Mixed models analyses

**FIGURE 1:**
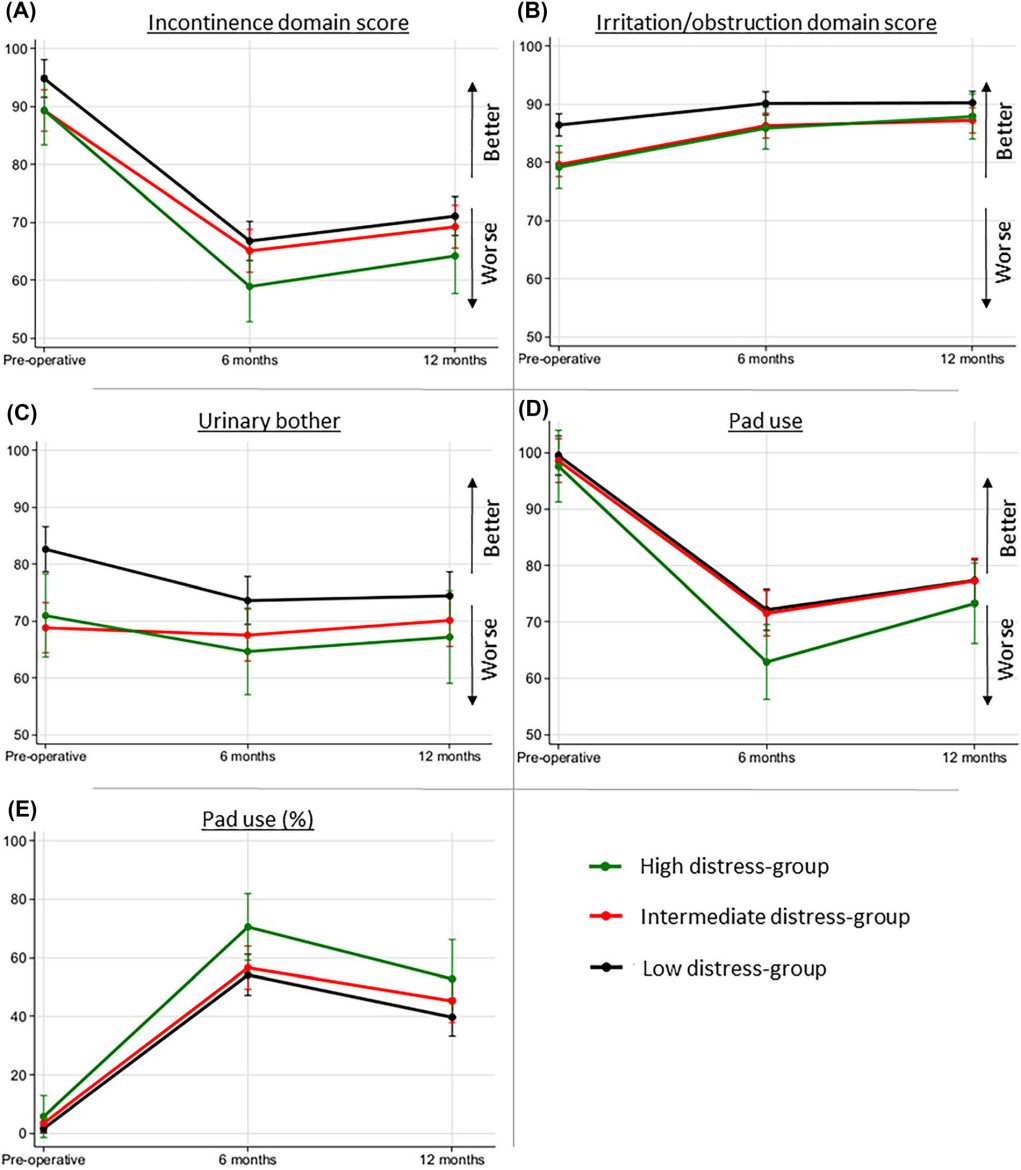
Mixed model analyses of urinary adverse effects.

**FIGURE 2:**
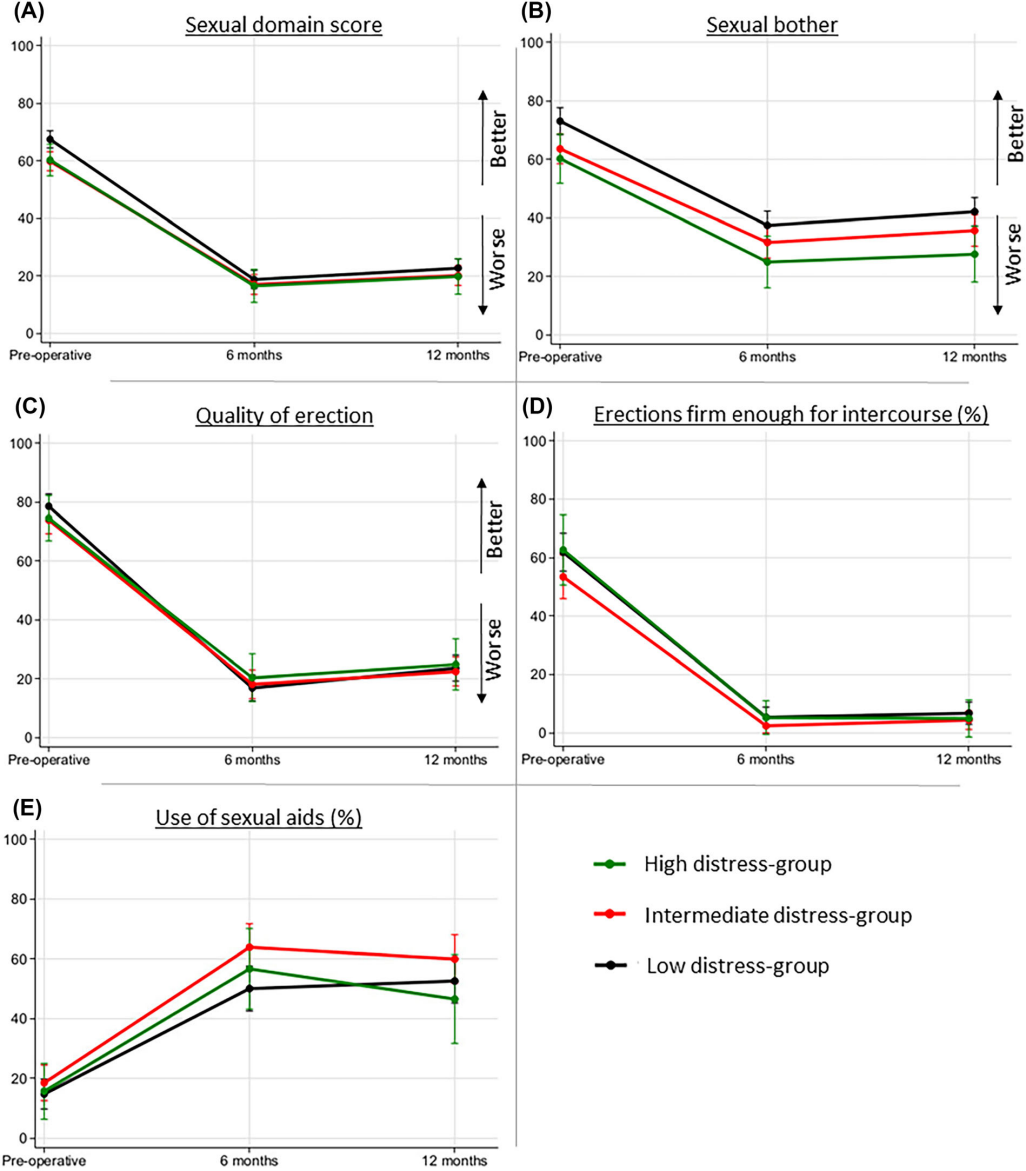
Mixed model analyses of sexual adverse effects.

Figures 1 and 2 show the results of the mixed models analyses and Table 3 the results from the post hoc pairwise tests between the distress groups.

**TABLE 3:** Pairwise tests between the low‐ and intermediate‐distress groups and low‐ and high‐distress groups at different time points before and after radical prostatectomy, based on the estimated coefficients obtained from linear mixed models.

Variable	Low distress versus intermediate distress	Low distress versus high distress
Incontinence score		
Baseline	**5.5 (0.025)**	5.5 (0.11)
6 months	1.7 (0.51)	**7.9 (0.028)**
12 months	1.8 (0.47)	6.9 (0.07)
Irritation/obstruction score		
Baseline	**6.8 (<0.001)**	**7.3 (0.001)**
6 months	**3.8 (0.011)**	**4.3 (0.043)**
12 months	**3.0 (0.044)**	2.4 (0.287)
Urinary bother		
Baseline	**13.8 (<0.001)**	**11.6 (0.006)**
6 months	6.1 (0.055)	**9.0 (0.044)**
12 months	4.3 (0.17)	7.3 (0.12)
Pad use		
Baseline	0.9 (0.73)	1.9 (0.61)
6 months	0.6 (0.82)	**9.3 (0.017)**
12 months	0.1 (0.98)	4.1 (0.32)
Use of one or more pads (cat.)		
Baseline	−1.7% (0.26)	−4.0% (0.15)
6 months	−2.4% (0.63)	**−16.4% (0.034)**
12 months	−5.5% (0.27)	−13.1% (0.101)
Sexual score		
Baseline	**7.6 (0.001)**	**7.2 (0.024)**
6 months	1.7 (0.48)	2.2 (0.52)
12 months	2.5 (0.29)	2.9 (0.42)
Sexual bother		
Baseline	**9.5 (0.007)**	**12.8 (0.009)**
6 months	5.9 (0.11)	**12.5 (0.015)**
12 months	6.4 (0.08)	**14.5 (0.008)**
Erection		
Baseline	4.7 (0.15)	4.1 (0.37)
6 months	1.2 (0.71)	3.5 (0.46)
12 months	1.2 (0.73)	1.3 (0.80)
Erection enough for intercourse (cat.)		
Baseline	8.5% (0.10)	−0.7% (0.91)
6 months	2.9% (0.19)	0.1% (0.97)
12 months	2.3% (0.37)	1.8% (0.66)
Use of sexual aids (cat.)		
Baseline	−3.7% (0.34)	−0.9% (0.86)
6 months	**−13.8% (0.015)**	−6.6% (0.41)
12 months	−7.3% (0.20)	6.1% (0.48)

*Note*: Absolute differences for scores are given for continuous variables and absolute differences in proportion (%) for categorical variables. Corresponding *p* values in parentheses. Statistically significant differences (*p* < 0.05) are highlighted with bold typeface.

#### 3.2.1 Urinary AEs

The analysis of urinary incontinence domain score showed that the low‐distress group had better scores compared with the intermediate and high groups at baseline (94.8 vs. 89.3 and 89.3, *p* = 0.025 and *p* = 0.11, Figure 1A). The scores of all groups decreased at 6 months with a small recovery at 12 months. The high‐distress group had a steeper decline than the other groups and lower scores at both 6 and 12 months (high vs. low; 58.9 vs. 66.8, *p* = 0.028 and 64.2 vs. 71.1, *p* = 0.07, Figure 1A).

At baseline, the low‐distress group had significantly less symptoms of urinary irritation and obstruction than the intermediate‐ and high‐distress groups (86.5 vs. 79.6 and 79.2, *p* < 0.001 and *p* = 0.001, Figure 1B). The scores of all three groups improved at follow‐up, but the low‐distress group still had less symptoms compared with the two other groups (low vs. intermediate; 90.2 vs. 86.3, *p* = 0.011 at 6 months and 90.3 vs. 87.2, *p* = 0.044 at 12 months, Figure 1B).

The intermediate‐ and high‐distress groups had more urinary bother at baseline than the low‐distress group (high vs. low; 71.0 vs. 82.6, *p* = 0.006, Figure 1C). During follow‐up, the scores of the intermediate‐distress group were stable, while those of the high‐ and low‐distress groups decreased moderately at 6 months with a small recovery at 12 months for the high‐distress group. The low‐distress group had less bother than the other groups also at follow‐up (low vs. high; 73.6 vs. 64.7, *p* = 0.044 at 6 months and 74.4 vs. 67.2, *p* = 0.12 at 12 months, Figure 1C).

Few used urinary pads at baseline, and there were no differences between the distress groups (Figure 1D). At follow‐up, more pads were used in the high‐distress group, although statistical significance was not reached at 12 months (Figure 1D). At 6 months, the high‐distress groups had a 16.4% points increased risk of using pads compared with the low‐distress group (high vs. low; 70.6% vs. 54.2%, *p* = 0.034 at 6 months and 52.8% vs. 39.7%, *p* = 0.10 at 12 months, Figure 1E).

#### 3.2.2 Sexual AEs

At baseline, the low‐distress group had a higher sexual domain score than the intermediate‐ and high‐distress groups (low vs. intermediate; 67.5 vs. 59.8, *p* = 0.001, Figure 2A). At 6 months, there was a steep decline in all groups. The scores converged and there were no longer any differences between groups (low vs. high 18.8 vs. 16.6, *p* = 0.52 at 6 months, Figure 2A). There were negligible improvement at 12 months in all groups (low vs. high; 22.7 vs. 19.8, *p* = 0.42, Figure 2A).

During all time points, the low‐distress group had less sexual bother than the intermediate‐ and high‐distress groups (low vs. high; 73.1 vs. 60.2, *p* = 0.009 at baseline, Figure 2B). Sexual bother increased at 6 months for all groups with a slight recovery at 12 months (low vs. high; 37.5 vs. 24.9, *p* = 0.015 at 6 months and 42.1 vs. 27.6, *p* = 0.008 at 12 months, Figure 2B). Results indicate a persistent difference in sexual bother between distress groups during follow‐up.

There was no difference between distress groups regarding quality of erections at baseline (Figure 2C,D). The scores dropped dramatically for all groups at 6 months with a small improvement at 12 months, but few had erections good enough for intercourse postoperatively (high vs. low; 5.0% vs. 6.8%, *p* = 0.66 at 12 months, Figure 2D). There were no differences between distress groups at any time points.

Around one out of six of the participants had used sexual aids at baseline, this increases to more than half of the patients at follow‐up (Figure 2E). The intermediate group had a higher probability of using sexual aids, with 13.8% points increased risk comparing to the low group at 6 months (intermediate vs. low; 63.8% vs. 50.0%, *p* = 0.015 at 6 months and 59.9% vs. 52.6% at 12 months, *p* = 0.20, Figure 2E).

### 3.3 Use of medications

The sub‐analysis of the 203 participants from Telemark on the use of medications showed that 74 (36%) men used antihypertensives, 29 (14%) used β‐blockers, 28 (14%) used α‐blockers/5‐α reductase inhibitors and three (1%) used antimuscarinics or β3‐stimulators (Table S2). Three (1%) men used selective serotonin reuptake inhibitors, two (1%) tricyclic antidepressants and one man used antipsychotics. Eleven (5%) men used hypnotica and six (3%) benzodiazepines, and the use of these medications was more common among the high‐ and intermediate‐distress groups (*p* = 0.003). Otherwise, there were no differences between groups regarding use of medications (Table S2).

## 4 DISCUSSION

This study showed that men with higher pre‐diagnostic levels of psychological distress had worse urinary incontinence, more urinary and sexual bother and more use of incontinence pads and sexual aids after RP, compared with men with lower pre‐diagnostic levels of psychological distress. Additionally, men with higher distress had more urinary and sexual symptoms already before diagnosis.

RP had overall a considerable negative effect on urinary incontinence, sexual function and urinary and sexual bother, as shown in multiple previous studies. Noteworthy, the differences in symptoms between the distress groups were often larger before diagnosis than after treatment. The difference in urinary incontinence at 6 months follow‐up was within the range (6–9 points) of defined MIDs.^20^ Regarding the sexual domain score, there was no difference between distress groups at follow‐up which might reflect the poor overall results of this domain. In this study, unlike previous reports, we chose to highlight the bother items of the EPIC‐26. Consequently, we found that more distressed patients seem more troubled by their lack of function, illustrated by the inferior sexual bother score among these men.

In line with our results, Mohamed et al. found that levels of anxiety and depression before treatment was a predictor for urinary and sexual dysfunction 6 months after PCa treatment.^11^ However, only 146 RP patients were included, and the generalizability of the results was questioned. Moreover, no items directly measuring urinary incontinence were used in their study.

In the longitudinal study from Punnen et al., preoperative anxiety and depression scores were associated with worse urinary and sexual function after RP.^12^ In contrast to our study, they had separate questionnaires for anxiety and depression and an additional 10‐grade visual analogue scale to rate emotional distress. Interestingly, only elevated baseline anxiety scores and not depression scores were associated with worse urinary EPIC scores at follow‐up, while both anxiety and depression were associated with worse sexual scores. When comparing with our study, there is an obvious risk of sampling bias in Punnen et al.'s study as only 13% to 30% of participants returned questionnaires at the 1–3 years follow‐up, and only 177 men were included in the final analysis. Lastly, while they regarded the differences as small and of uncertain clinical significance, we found differences in the range of MID for urinary incontinence.

Similar to our study, in the large retrospective analysis of Pompe et al., a short questionnaire was used to stratify men into distress groups.^14^ In agreement with our findings, men with higher preoperative levels of anxiety and depression more often used incontinence pads. Likewise, there were no differences in erectile recovery between the groups. However, the more distressed men used more PDE5 inhibitors at follow‐up, while we found a higher use of sexual aids. Contrary to our study, Pompe et al.'s outcomes were purely functional and did not address how participants felt or were bothered by their symptoms. Furthermore, while our study was population based and all RP patients were included, Pompe et al. excluded patients receiving non‐nerve‐sparing procedures, postoperative radiation and/or androgen‐deprivation therapy, which is important when comparing results. Finally, Pompe et al. pointed out the lack the lack of information on patients' medications as a study limitation, while our study included such information.

Patients with anxiety and depression generally report a higher symptom burden from chronic illnesses, also after controlling for the severity of the chronic disease.^26^ Correspondingly, in patients with functional urological disorders such as overactive bladder, personality and psychiatric comorbidities are associated with severity of symptoms.^27^ A recent large review and meta‐analysis found that men with depression had a more than threefold higher likelihood of experiencing LUTS than men without depression.^28^ There is also evidence for a strong association between depression and sexual dysfunction and men with depression have a 39% increased risk of erectile dysfunction compared with those without depression according to a previous meta‐analysis.^29^, ^30^ Possible mechanisms for these associations could be the effect of LUTS on the psychological status, for example, through sleeping deficiency or impeding social interaction.^28^ Moreover, there may be an influence of depression as such or the treatment of depression on hormonal balances, for example, diuretic hormones, catecholamine and testosterone levels, which in turn can affect both urinary and sexual function. Lastly, depression, LUTS and erectile dysfunction are thought to share the same pathological pathways, and LUTS and erectile dysfunction can in that context be seen as markers of depression.^28^, ^29^

The above mechanisms may explain the present differences between distress groups at baseline identified in this study and are probably relevant to the AEs seen after RP. Furthermore, more distressed patients might be more worried about small leakages, making them reluctant to omit safety pads, which could explain a higher use of pads. Similarly, worrying about how erectile dysfunction and lack of intimacy will affect the relationship to one's partner could lead to a higher use of sexual aids. On the other hand, high distress could negatively affect adherence to follow‐up and rehabilitation programmes, leaving AEs inadequately treated.

Our study has some limitations. The HSCL‐5 is recommended for screening purposes and cannot replace the ordinary work‐up to diagnose anxiety and depression. Moreover, because anxiety and depression are known to be highly correlated, both constructs should be seen as a single characteristic when using the HSCL‐5, and we have therefore not differentiated between the two.^22^ Although a cut‐off of HSCL‐5 = 2 has previously been proposed to identify psychiatric conditions and defines our high‐distress group, the stratification of HSCL‐5 scores into three groups is not described before, which could question the validity of our results. On the other hand, we believe this stratification is quite straightforward because the low‐distress group does not report any distress at all and the intermediate‐distress group are all participants in between high and low distress.

In our analyses, we chose not to adjust for post‐diagnostic variables like nerve‐sparing surgery and adjuvant treatment. The rationale for this was that unlike iPSA, these factors were unknown for the patient at the time of filling out the baseline questionnaire. To estimate the true effect of psychological distress at that time, post‐diagnostic factors were omitted.

Although the inclusion rate was high, some bias in the estimated associations due to differences in non‐response between groups cannot be completely ruled out. For instance, we know nothing about the distress levels of those who did not accept the study invitation. Even though there was a higher iPSA among the 5% dropouts, there is no plausible reason why this should alter the general conclusions of the study. A higher completion rate in the high‐distress group at 12 months might strengthen our findings and add to the statistical significance at this time point. Although the sub‐analysis of a little less than half of the patients showed that apart from that the high‐distress group used more benzodiazepines and hypnotics, there were no differences between groups in the use of other medications. We cannot dismiss associations entirely, but given the overall use of medications was low, it is most likely not an important confounder that would change the overall results in a complete analysis. Furthermore, the higher use of tranquillizers among the more distressed corroborated the validity of HSCL‐5.

The strengths of our study are the prospective design, the use of well‐validated questionnaires to measure distress and outcome, the population‐based setting with high inclusion and completion rate and the adjustment for relevant confounders. To our knowledge, this is the first longitudinal study of the association between distress and AEs after RP to measure baseline distress before diagnosis instead of between diagnosis and treatment. Although the referred men at that time point might have been aware of that they had an elevated PSA, they were still not diagnosed with PCa. We argue that the baseline or our study better represent the general level of distress of the participants rather than the reaction from being newly diagnosed with cancer or concerns about the impending surgery.

We believe that the findings of this study are important for both urologists and for men with newly diagnosed PCa where definitive treatment is an option. Although the presence of anxiety or depression in a patient's history most of the time will not be decisive for what treatment to choose, this new knowledge could be one factor among others to consider. Furthermore, men with elevated levels of distress may need intensified rehabilitation after the surgery has been performed, and future studies should focus on optimizing preoperative and postoperative counselling and follow‐up for these men.

## 5 CONCLUSIONS

Men with high levels of anxiety and depression report more urinary and sexual AEs after RP. This new knowledge should be considered when deciding on treatment after diagnosis of non‐metastatic PCa and during follow‐up after RP.

## AUTHOR CONTRIBUTIONS

*Study concept and design*: Rasmus Nilsson, Tor Åge Myklebust, Thomas Næss‐Andresen, Tomm Bernklev, Hege Kersten, and Erik Skaaheim Haug. *Acquisition of data*: Rasmus Nilsson, Thomas Næss‐Andresen, and Erik Skaaheim Haug. *Analysis and interpretation of data*: Rasmus Nilsson, Tor Åge Myklebust, and Erik Skaaheim Haug. *Drafting of the manuscript*: Rasmus Nilsson, Thomas Næss‐Andresen, Tomm Bernklev, Hege Kersten, Tor Åge Myklebust, and Erik Skaaheim Haug. *Critical revision of the manuscript for important intellectual content*: Rasmus Nilsson, Thomas Næss‐Andresen, Tomm Bernklev, Hege Kersten, Tor Åge Myklebust, and Erik Skaaheim Haug. *Statistical analysis*: Rasmus Nilsson and Tor Åge Myklebust. *Obtaining funding*: Rasmus Nilsson, Tomm Bernklev, Hege Kersten, and Erik Skaaheim Haug. *Administrative, technical, or material support*: Rasmus Nilsson, Thomas Næss‐Andresen, Tomm Bernklev, Hege Kersten, and Erik Skaaheim Haug. *Supervision*: Tomm Bernklev, Hege Kersten, Tor Åge Myklebust, and Erik Skaaheim Haug.

## CONFLICT OF INTEREST STATEMENT

The authors declare no conflicts of interest.

## Supporting information


**Table S1.** Attrition analysis.
**Table S2.** Medication at baseline.

## Data Availability

Due to privacy protection rules, the data cannot be publicly shared.
