# Supplementary material for: The association between pre‐diagnostic levels of psychological distress and adverse effects after radical prostatectomy
Source: BJUI Compass. 2024 Feb 24;5(5):502–11. doi: 10.1002/bco2.334 (PMC11090769; doi:10.1002/bco2.334)
Supplement: Supplementary file 1 — Table S1. Attrition analysis. Table S2. Medication at baseline. [file BCO2-5-502-s001.docx]

Supplements

Table S1. Attrition analysis

| **Variables** | **Not returning follow-up questionnaires**  **N=19** | **Returning at least one follow-up questionnaire**  **N=397** | **p-value** |
| --- | --- | --- | --- |
| HSCL-score at baseline, mean (SD) | 1.45 (0.45) | 1.33 (0.45) | 0.25 |
| Age (years) at diagnosis, mean (SD) | 64.8 (7.1) | 66.9 (6.3) | 0.17 |
| *Level of education, N(%)*  ≤12 years  >12 years | 11 (65)  6 (35) | 234 (59)  161 (41) | 0.65 |
| *BMI (kg/m²), mean (SD)* | 25.8 (2.9) | 27.1 (3.4) | 0.11 |
| *Psychical activity, N (%)*  Inactive  Minimally active  Highly active | 4 (21)  8 (42)  7 (37) | 41 (10)  161 (41)  193 (49) | 0.29 |
| *Comorbidities, N (%)*  0-1  2 or more | 17 (90)  2 (10) | 291 (73)  106 (27) | 0.11 |
| PSA at biopsy (µg/L), mean (SD) | 15.9 (11.2) | 10.0 (7.5) | **0.001** |
| Urinary incontinence score, mean (SD) | 93.8 (15.3) | 91.8 (13.6) | 0.57 |
| Urin. irritation/obstruction score, mean (SD) | 82.9 (18.5) | 82.5 (15.8) | 0.91 |
| Sexual domain score, mean (SD) | 65.1 (25.2) | 63.7 (27.8) | 0.84 |
| Postoperative radiotherapy, N (%) | 4 (21) | 35 (9) | 0.07 |
| Postoperative hormonal treatment, N (%) | 1 (5) | 31 (8) | 0.68 |

Table S2. Medication at baseline

| **Medication N(%)**  **(ATC-codes)** | **Low distress**  **(SCL-5 = 1)**  **N=94** | **Intermediate distress**  **(SCL-5 = 1.2-1.8)**  **N=78** | **High distress**  **(SCL-5 = 2 or higher)**  **N=31** |
| --- | --- | --- | --- |
| Antimuscarinics or β3-stimulators  (G04BD, G04BC) | 2 (2) | 1 (1) | 0 (0) |
| α-blockers/5-α reductase inhibitors  (G04CA, G04CB) | 12 (13) | 13 (17) | 3 (10) |
| β-blockers  (C07A, C07B) | 11 (12) | 13 (17) | 5 (16) |
| Antihypertensives  (C02, C03, C08, C09) | 34 (36) | 27 (35) | 13 (42) |
| Acetylsalisylic-acid  (B01AC06) | 18 (19) | 16 (21) | 8 (27) |
| Selective Serotonin Reuptake Inhibitors  (N06AB) | 1 (1) | 1 (1) | 1 (3) |
| Hypnotics  (N05CF01, N05CF02) | 2 (2) | 4 (5) | 5 (16) |
| Benzodiazepines  (N05B, N05CD) | 0 (0) | 2 (3) | 4 (13) |
| Tricyclic antidepressants  (N06A) | 0 (0) | 1 (1) | 1(3) |
| Antipsychotics  (N05A) | 0 (0) | 1 (1) | 0 (0) |

During follow-up, 8 patients started with antimuscarinics or B3 stimulators. Two in the low-, 5 in the intermediate- and 1 in the high-distress group.
